# Supplementary material for: DNA sequencing, microbial indicators, and the discovery of buried kimberlites
Source: Commun Earth Environ. 2023 Oct 21;4(1):387. doi: 10.1038/s43247-023-01020-z (PMC11041713; doi:10.1038/s43247-023-01020-z)
Supplement: Supplementary file 1 — Supplementary Information [file 43247_2023_1020_MOESM1_ESM.pdf]

Supplementary Information:

DNA sequencing, microbial indicators, and the discovery of buried kimberlites

Rachel L. Simister[1,2], Bianca P. Iulianella Phillips [2,3], Andrew P. Wickham[2,3], Erika M. Cayer[2,3], Craig J.R. Hart [2,3], Peter A. Winterburn [2,3]† and Sean A. Crowe [1, 2] *

[1]Department of Microbiology & Immunology, University of British Columbia, Vancouver, BC, V6T 1Z3, Canada

[2]Department of Earth, Ocean and Atmospheric Sciences, University of British Columbia, Vancouver, BC, V6T 1Z4, Canada

[3]MDRU-Mineral Deposit Research Unit, Department of Earth, Ocean and Atmospheric Sciences, University of British Columbia, Vancouver, BC, V6T 1Z4, Canada

† Deceased

* Corresponding author

Supplemental Figures and Legends

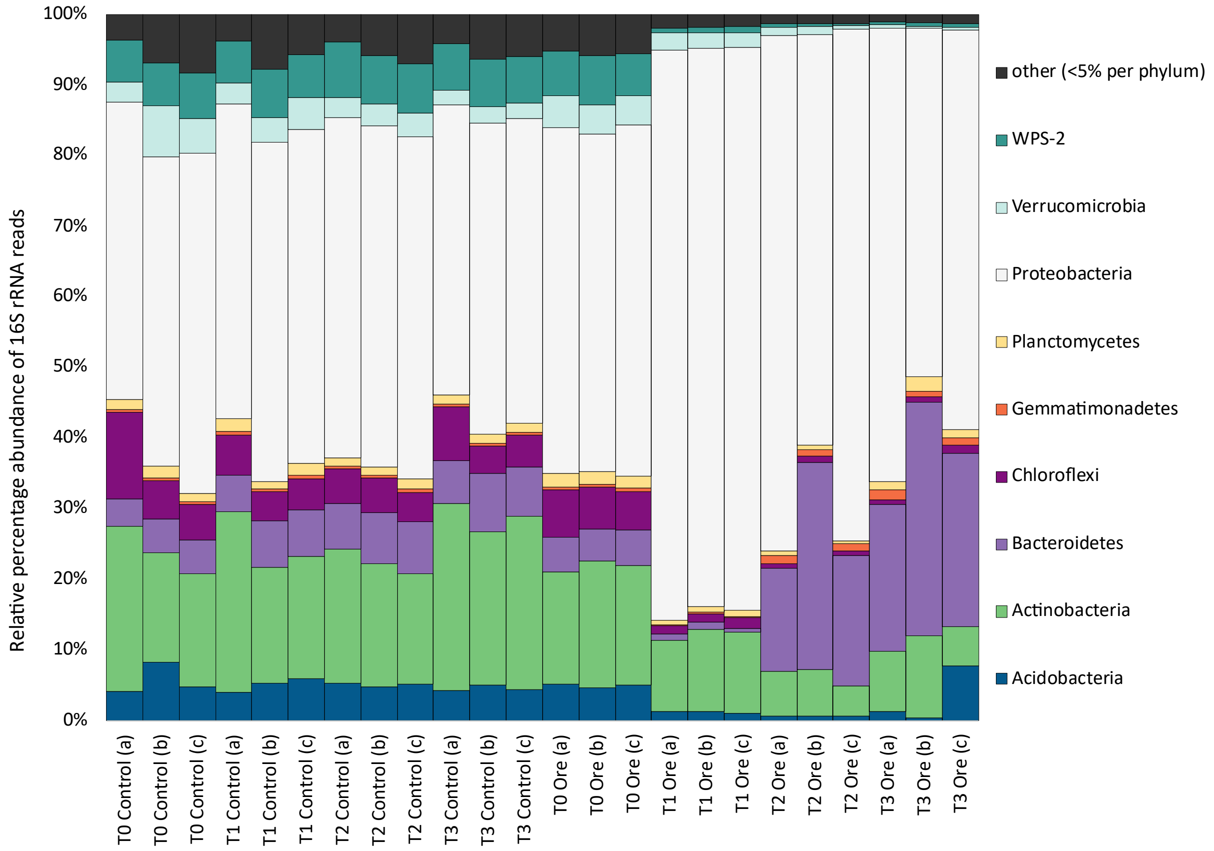

Supplemental Figure 1: Distribution of 16S rRNA reads per phylum for each sample. The number of reads per phylum is calculated as a percentage of the total reads for each sample. The '*other' grouping represents summed phyla that individually contributed <5% of the total number of reads per sample.

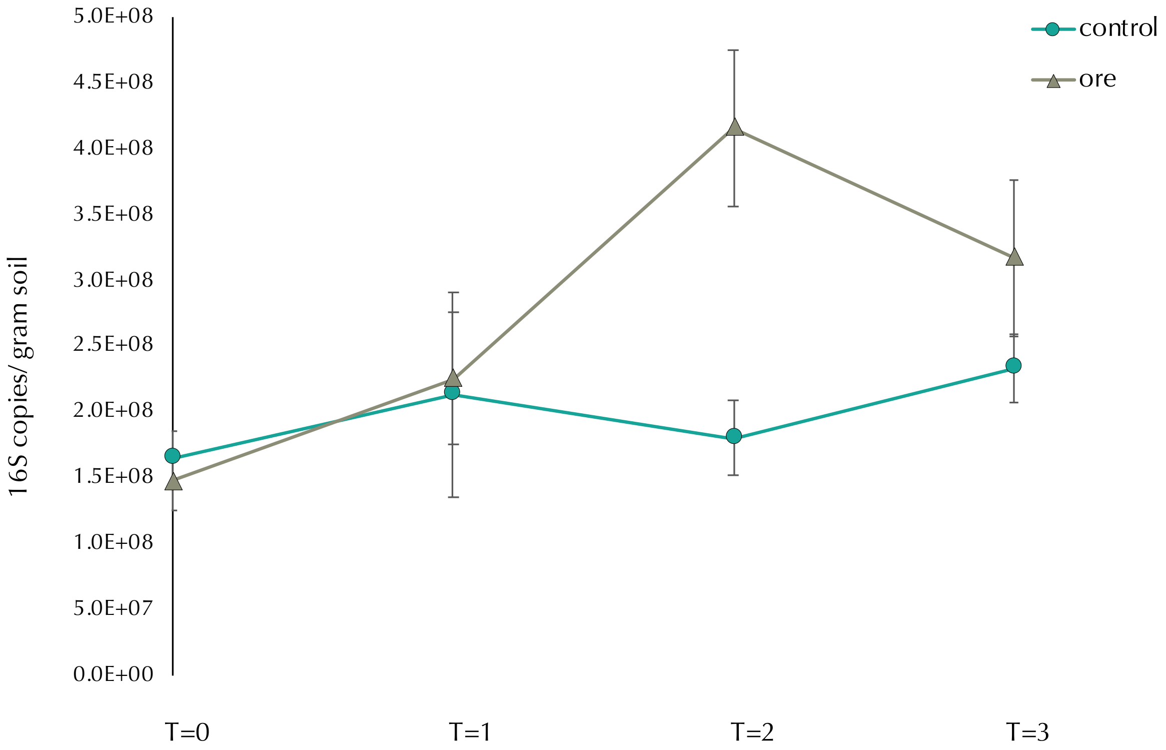

Supplemental Figure 2: qPCR of 16S rRNA copies per gram of soil from incubation experiments.

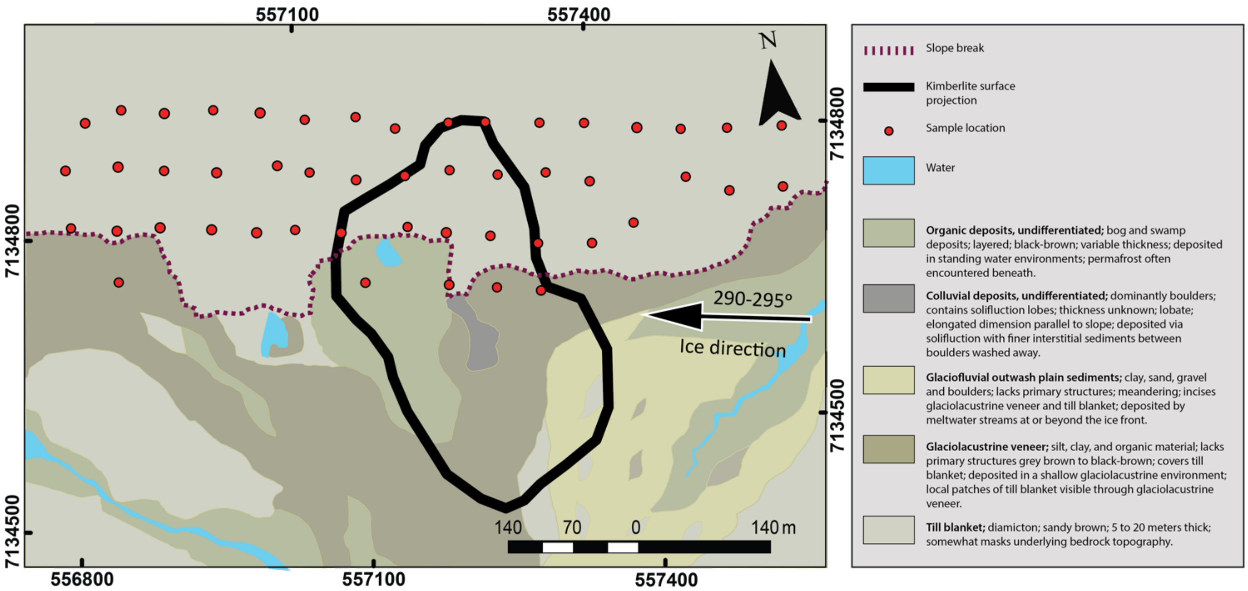

Supplemental Figure 3a: Sample locations for DO-18 soil/till samples (red circles) overlying a surficial materials map derived from field observations.

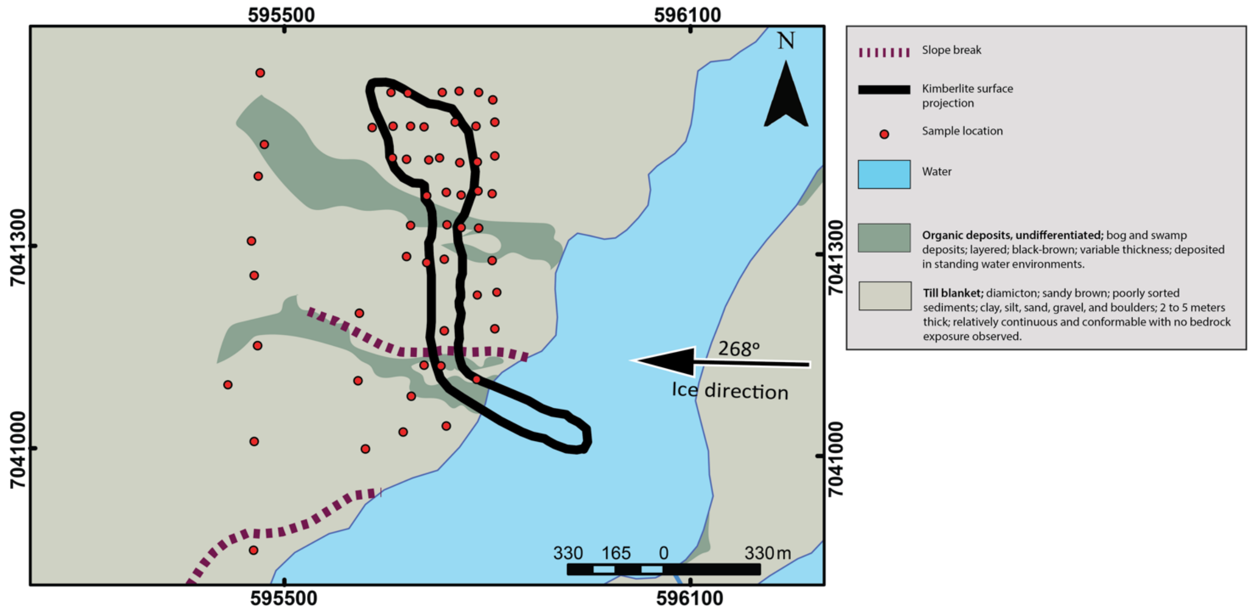

Supplemental Figure 3b: Sample locations for Kelvin soil/till samples (red circles) overlying a surficial materials map derived from field observations.

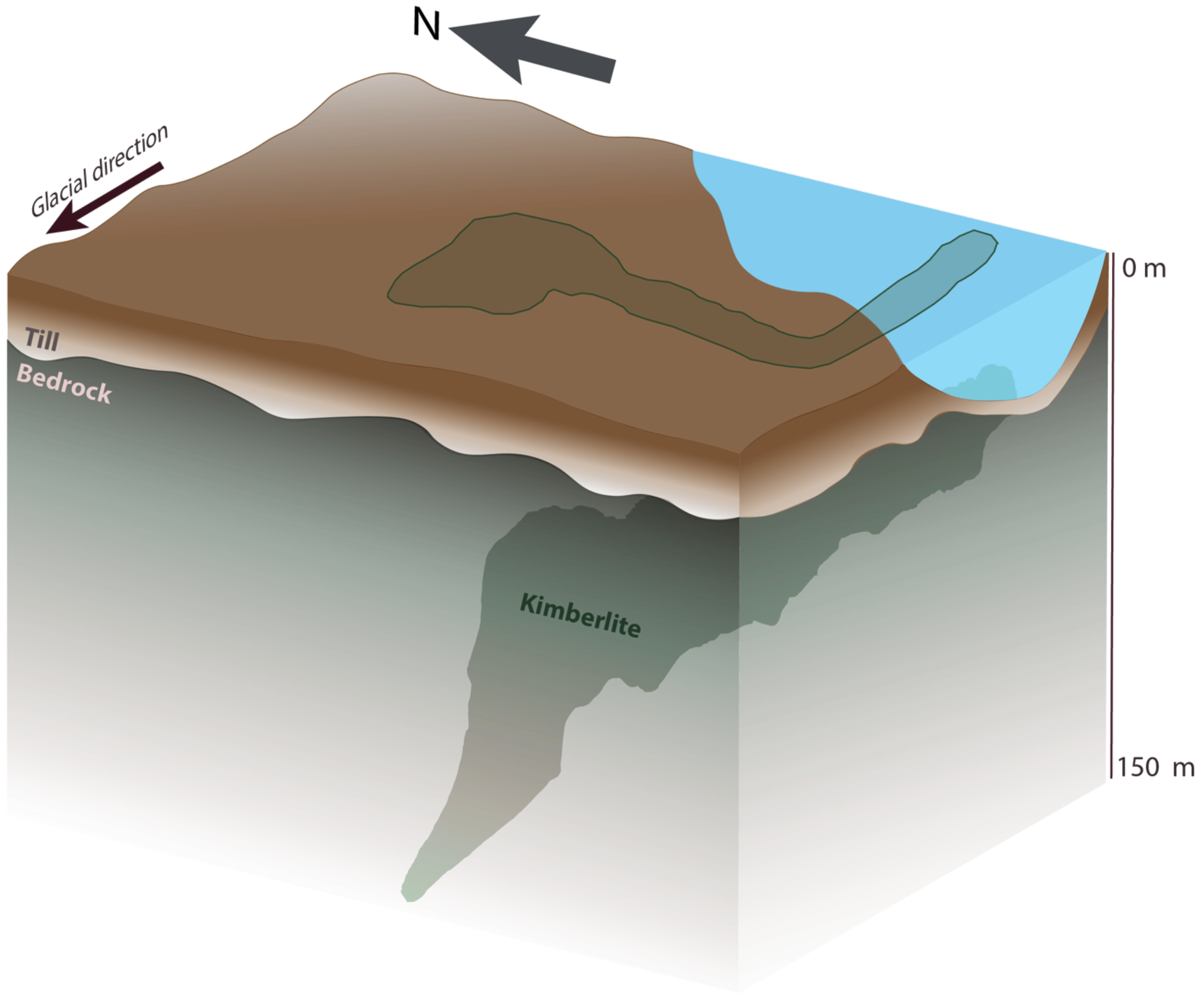

Supplemental Figure 3c: Surface projection and three-dimensional block model of the L-shaped Kelvin kimberlite emplaced into metaturbidite bedrock and covered by glacial till (~4 m), illustrating the thickness of rock and overburden present above the kimberlite. The geometry and depth of kimberlite emplacement at Kelvin is based on drilling and modelling described by Bezzola et al.[1].

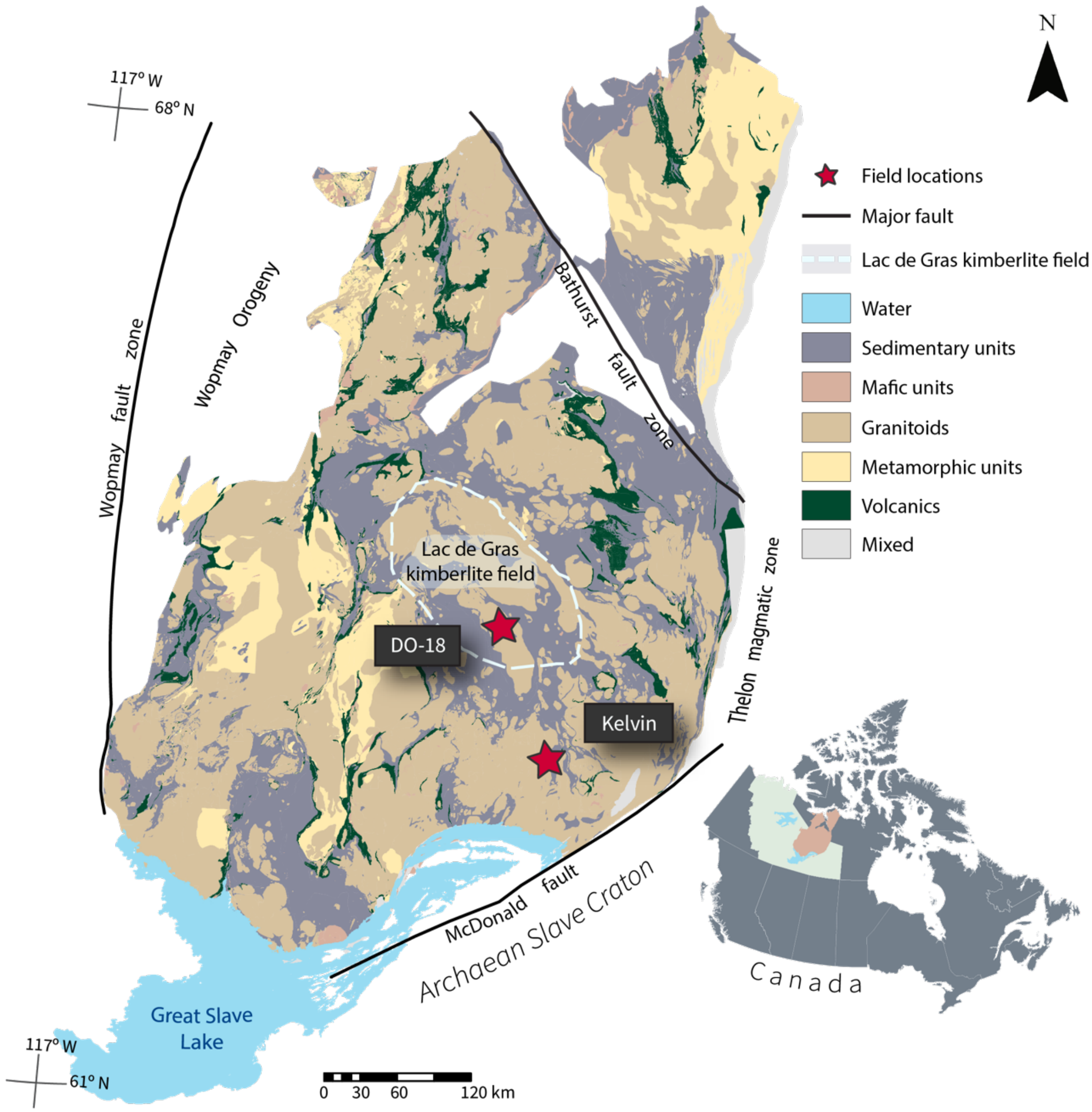

Supplemental Figure 4: Simplified geologic map of the Archean Slave craton, Northwest Territories, with the DO-18 and Kelvin kimberlites indicated with red stars. Thick black lines represent major faults/fault systems. Geological units are based on bedrock maps made publicly available from the Northwest Territories Geological Survey (NWT Open file 2019-01)[2].

Supplementary References

1. Bezzola, M., et al., Geology and resource development of the Kelvin kimberlite pipe, Northwest Territories, Canada. *Mineralogy and Petrology*. **112**(2): p. 463-475 (2018).

2. Stubley, M. and Irwin, D. Bedrock Geology of the Slave Craton, Northwest Territories and Nunavut; Northwest Territories Geological Survey. *NWT Open File 2019-01* (2019).
