# Supplementary material for: DNA sequencing, microbial indicators, and the discovery of buried kimberlites
Source: Commun Earth Environ. 2023 Oct 21;4(1):387. doi: 10.1038/s43247-023-01020-z (PMC11041713; doi:10.1038/s43247-023-01020-z)
Supplement: Supplementary file 14 — Description of Additional Supplementary Files [file 43247_2023_1020_MOESM14_ESM.pdf]

Description of Additional Supplementary Files

Supplemental Data 1: Geochemical data for kimberlite and soil used in amendments. For calculating the % increase of each analyte in the soil amendment where soil or kimberlite concentrations were below the detection limit, one half of the detection limit was used (e.g., <0.1 = 0.05).

Supplemental Data 2: Incubation experiment. Overview of the species estimates and diversity metrics obtained per sample after quality filtering. Abbreviation: OTU, operational taxonomic unit.

Supplemental Data 3a: Indicators for kimberlite amendment.

Supplemental Data 3b: Indicators for control.

Supplemental Data 4: The abundance of the top 20 most abundant OTUs in DO18 and kelvin field samples.

Supplemental Data 5: DO-18 field samples. Overview of the species estimates and diversity metrics obtained per sample after quality filtering. Abbreviation: OTU, operational taxonomic unit.

Supplemental Data 6: Kelvin field samples. Overview of the species estimates and diversity metrics obtained per sample after quality filtering. Abbreviation: OTU, operational taxonomic unit.

Supplemental Data 7: Averaged summed % relative abundance for "on" and "off" samples at field locations.

Supplemental Data 8a: Geochemical metadata for DO-18. Geochemical data digestion codes for ALS Mineral Laboratories Ltd.: multi-acid digestion (4-acid) - ME-MS61L.

Supplemental Data 8b: Geochemical metadata for Kelvin. Geochemical data digestion codes for ALS Mineral Laboratories Ltd.: multi-acid digestion (4-acid) - ME-MS61L.

Supplemental Data 9: Response (RR) for randomization tests.
