# Supplementary material for: DNA sequencing, microbial indicators, and the discovery of buried kimberlites
Source: Commun Earth Environ. 2023 Oct 21;4(1):387. doi: 10.1038/s43247-023-01020-z (PMC11041713; doi:10.1038/s43247-023-01020-z)
Supplement: Supplementary file 15 — Reporting Summary [file 43247_2023_1020_MOESM15_ESM.pdf]

nature portfolio

Corresponding author(s): Sean A Crowe

Last updated by author(s): May 26th 2023

Reporting Summary

Nature Portfolio wishes to improve the reproducibility of the work that we publish. This form provides structure for consistency and transparency in reporting. For further information on Nature Portfolio policies, see our Editorial Policies and the Editorial Policy Checklist.

Statistics

For all statistical analyses, confirm that the following items are present in the figure legend, table legend, main text, or Methods section.

n/a	Confirmed	
☐	☒	The exact sample size (n) for each experimental group/condition, given as a discrete number and unit of measurement
☐	☒	A statement on whether measurements were taken from distinct samples or whether the same sample was measured repeatedly
☐	☒	The statistical test(s) used AND whether they are one- or two-sided<br>*Only common tests should be described solely by name; describe more complex techniques in the Methods section.*
☐	☒	A description of all covariates tested
☐	☒	A description of any assumptions or corrections, such as tests of normality and adjustment for multiple comparisons
☐	☒	A full description of the statistical parameters including central tendency (e.g. means) or other basic estimates (e.g. regression coefficient) AND variation (e.g. standard deviation) or associated estimates of uncertainty (e.g. confidence intervals)
☐	☒	For null hypothesis testing, the test statistic (e.g. F, t, r) with confidence intervals, effect sizes, degrees of freedom and P value noted<br>*Give P values as exact values whenever suitable.*
☒	☐	For Bayesian analysis, information on the choice of priors and Markov chain Monte Carlo settings
☒	☐	For hierarchical and complex designs, identification of the appropriate level for tests and full reporting of outcomes
☐	☒	Estimates of effect sizes (e.g. Cohen's d, Pearson's r), indicating how they were calculated

Our web collection on statistics for biologists contains articles on many of the points above.

Software and code

Policy information about availability of computer code

Data collection	no software was used for data collection
Data analysis	used open source tool for data analysis: Schloss PD et al. 2009. Introducing mothur: Open-source, platform-independent, community-supported software for describing and comparing microbial communities. Applied and Environmental Microbiology 75:7537–7541.

For manuscripts utilizing custom algorithms or software that are central to the research but not yet described in published literature, software must be made available to editors and reviewers. We strongly encourage code deposition in a community repository (e.g. GitHub). See the Nature Portfolio guidelines for submitting code & software for further information.

Data

Policy information about availability of data

All manuscripts must include a data availability statement. This statement should provide the following information, where applicable:

- Accession codes, unique identifiers, or web links for publicly available datasets
- A description of any restrictions on data availability
- For clinical datasets or third party data, please ensure that the statement adheres to our policy

sequences were deposited into the Sequence read archive (SRA) under accession number PRJNA698256.

Research involving human participants, their data, or biological material

Policy information about studies with human participants or human data. See also policy information about sex, gender (identity/presentation), and sexual orientation and race, ethnicity and racism.

Reporting on sex and gender	not applicable
Reporting on race, ethnicity, or other socially relevant groupings	not applicable
Population characteristics	not applicable
Recruitment	not applicable
Ethics oversight	not applicable

Note that full information on the approval of the study protocol must also be provided in the manuscript.

Field-specific reporting

Please select the one below that is the best fit for your research. If you are not sure, read the appropriate sections before making your selection.

☐ Life sciences ☐ Behavioural & social sciences ☒ Ecological, evolutionary & environmental sciences

For a reference copy of the document with all sections, see nature.com/documents/nr-reporting-summary-flat.pdf

Ecological, evolutionary & environmental sciences study design

All studies must disclose on these points even when the disclosure is negative.

Study description	We evaluated the applicability of microbial community fingerprinting when exploring for diamondiferous kimberlites concealed by glacial overburden in northern latitudes. Soil samples were collected from orientation surveys at the DO-18 (49 samples) and Kelvin (55 samples) kimberlites in the Northwest Territories, Canada. The soil samples underwent 16S rRNA gene sequencing and differential abundance analyses to find indicator species that are representative of subsurface kimberlite deposits.
Research sample	At each sampling station 1 kg of B-horizon soil was collected for geochemical analyses (4-acid digests with ICP-MS finish) and ~200 g for microbiological analyses (16S rRNA gene sequencing). B-horizon soils were chosen as they are the most commonly sampled horizon during soil-based mineral exploration surveys and because it is a geochemically relevant mineral horizon.
Sampling strategy	The sampling surveys were designed to sample across the surface projection of the kimberlites. Sample density and number of samples depended on the size and geometry of each kimberlite. Enough samples were taken to sufficiently pass over the target through to background material. Approximately 1/3 of the samples were intentionally located directly over the surface expression of the kimberlites and the remainder were located above background materials in both down-ice and up-ice directions.
Data collection	Field data (geologic observations, physicochemical variables, environmental observations, etc.) were recorded in waterproof notebooks in the field and transferred to computer each evening. Data collection and soil sample collection was performed by Erika Cayer (DO-18), Andy Wickham (Kelvin), and Bianca Iulianella Phillips (Kelvin).
Timing and spatial scale	Samples at DO-18 were collected over 15 days between July 9th, 2015, and July 23rd, 2015, prior to storage at -20ºC, and DNA extraction in 2017. Approximately 8-12 samples were collected each day. Samples at Kelvin were collected over 12 consecutive days between July 11th, 2017, and July 22nd, 2017, prior to storage at -20ºC, and DNA extraction in 2017. Approximately 30 samples were collected each day. The DO-18 survey was conducted over 500 m2 and the Kelvin survey was conducted over 1 km2.
Data exclusions	At DO-18, samples collected below a slope-break with depositional materials were excluded from the 16S rRNA gene sequencing. This was done to avoid appreciable sample heterogeneity.<br><br>At Kelvin, a subset of samples overtop of the kimberlite and above background materials were chosen from a large sample archive (~240 samples) for 16S rRNA gene sequencing due to cost.
Reproducibility	Two field sites were used in this study to test for reproducibility of our indicator species across different field areas. This test was successful with shared anomalies that spatially correlate to the surface projection of the kimberlite bodies. Field duplicates (no greater than 3 m from the original sample) and certified reference materials (CRMs) were inserted into the sampling stream at 15%, for QA/QC of geochemical samples.
Randomization	Samples were allocated to either "above kimberlite/ON" or "above background/OFF". This is based on sample location with respect to the surface projection of the concealed kimberlite bodies. Kimberlite geometry is defined by geological drilling.
Blinding	Each soil sample was collected in the same way regardless of the composition of the underlying geology. Sample allocation was

Blinding	dependent on pre-set parameters (proximity to kimberlite body).

Did the study involve field work? ☒ Yes ☐ No

Field work, collection and transport

Field conditions	Sampling was performed in the summertime with temperatures fluctuating between 18 and 25 ºC. Rain was light and intermittent over sampling duration in both cases.
Location	DO-18: 64°20'03.9"N 109°48'36.7"W<br><br>Kelvin: 63°29'09.5"N 109°04'35.1"W
Access & import/export	Field work was facilitated in part by the Government of the Northwest Territories (Northwest Territories Geological Survey (NTGS)). Permits were not required as work was conducted under active mineral exploration permits held by the companies who hosted us on the property and in camp – Kennady Diamonds (Kelvin) and Peregrine Diamonds (DO-18) at the time of sampling, now Mountain Province Diamonds and De Beers, respectively. Soil samples were shipped to the University of British Columbia directly or to the analytical lab (ALS) via the NTGS. All shipping was domestic, and all samples were non-hazardous, requiring no special handling/care.
Disturbance	The fieldwork is considered low impact within the mineral exploration industry. Sample collection was performed on foot and no debris was left behind. Soil and the vegetation cap were placed back into the sampling hole to preserve the environment. Areas where there was evidence of animal habitation were avoided for sampling. Helicopter was only used at DO-18, when access was prohibited otherwise.

Reporting for specific materials, systems and methods

We require information from authors about some types of materials, experimental systems and methods used in many studies. Here, indicate whether each material, system or method listed is relevant to your study. If you are not sure if a list item applies to your research, read the appropriate section before selecting a response.

Materials & experimental systems

n/a	Involved in the study	
☒	☐	Antibodies
☒	☐	Eukaryotic cell lines
☒	☐	Palaeontology and archaeology
☒	☐	Animals and other organisms
☒	☐	Clinical data
☒	☐	Dual use research of concern
☒	☐	Plants

Methods

n/a	Involved in the study	
☒	☐	ChIP-seq
☒	☐	Flow cytometry
☒	☐	MRI-based neuroimaging
